# Pandemic preparedness in Vietnam: a review of health system resilience and areas for improvement

**DOI:** 10.7189/jogh.15.03001

**Published:** 2025-01-24

**Authors:** Bach Xuan Tran, Taufique Joarder, Vincent Junxiong Pang, Clara Marin Carballo, Hoa Thi Do, Cuong Tat Nguyen, Linh Gia Vu, Ngo Van Toan, Shenglan Tang

**Affiliations:** 1 Faculty of Public Health, VNU University of Medicine and Pharmacy, Vietnam National University, Hanoi, Vietnam; 2 SingHealth Duke-NUS Global Health Institute, National University of Singapore, Singapore; 3 Duke Global Health Institute, Duke University, North Carolina, USA; 4 Bloomberg School of Public Health, Johns Hopkins University, Baltimore, Maryland, USA; 5 Institute of Health Economics and Technology, Hanoi, Vietnam; 6 Institute for Global Health Innovations, Duy Tan University, Da Nang, Vietnam; 7 Scientific and Ethical Research Committee, Dong Nai University of Technology, Dong Nai, Vietnam

## Abstract

In this viewpoint, we explore Vietnam’s health system vulnerabilities and its national response to the COVID-19 pandemic, as well as critical areas of health system resilience, including health financing, workforce distribution, information systems, and governance. While Vietnam achieved early success through strong governance and mass vaccination campaigns, the pandemic revealed weaknesses in resource procurement, workforce imbalance, and limitations of its health information system. There are challenges in ensuring the rapid disbursement of financial resources and reliance on imported medical supplies, which delayed response times. Uneven healthcare workforce distribution, particularly in rural areas, further strained the health system. Although telemedicine and digital health solutions were implemented, weak digital infrastructure and inadequate information technology literacy hindered their effectiveness. Governance efforts, while generally strong, highlighted the need for better coordination and role clarity during health emergencies. Our findings identify areas for improvement, including effective resource mobilisation and allocation, enhanced digital infrastructure, expanded telemedicine access, and better support for healthcare workers. Governance enhancements, such as improved priority setting and interagency coordination, were also critical. These insights offer valuable guidance for strengthening Vietnam’s health system and ensuring greater preparedness for future pandemics, but could also help other low- and middle-income countries facing similar challenges.

The COVID-19 pandemic highlighted the vital need for robust health systems globally, with particular emphasis on low- and middle-income countries like Vietnam [1]. Although governments’ responsiveness to pandemic control has limited its impacts on population health, Vietnam's health system was severely tested as the pandemic evolved. The country’s success in overcoming these challenges is owed to collective efforts across various sectors, including the government, military, and public health, as well as whole-of-society efforts [2]. This solidarity was crucial in ensuring that, despite having limited resources, Vietnam managed to protect its population and maintain socioeconomic stability during a global health crisis.

However, there were several specific weaknesses in Vietnam’s health system during the pandemic that can be addressed for future pandemic preparedness [1]. While the country was lauded for its rapid and effective response, particularly during the initial stages, there is limited evidence of the systemic challenges that persisted, such as the increased strain on healthcare workers (HCWs) [3], especially in rural areas, and the difficulties in maintaining medical supply chains, which were at the time under unprecedented pressure. For instance, the Ministry of Health's struggle to meet the surging demand for vaccines highlights the need for more resilient and scalable infrastructure [2]. Addressing these gaps is essential, not only to better prepare Vietnam for future health crises, but also to provide a reference for other low- and middle-income countries facing similar vulnerabilities.

Health system resilience is defined as the capacity to prevent, prepare for, respond to, and recover from public health threats, while maintaining essential health services, even in vulnerable and conflict-affected settings [4]. Previous studies have identified some of the successes and failures in Vietnam's COVID-19 response [1,2,5–10]. The country’s strategy, which evolved from strict lockdowns to a more flexible approach involving widespread vaccination and the use of technology for contact tracing, has been well documented [1]. Yet many of these studies have focussed on the initial success of Vietnam’s pandemic response without critically examining the subsequent stages when the health system resilience was tested. This left a gap in our understanding of the full spectrum of challenges faced by the health system and how these could be mitigated in the future. Pandemic preparedness involves proactive planning by governments, health systems, and communities to prevent, detect, respond to, and recover from infectious disease outbreaks. Prior to writing this viewpoint, we reviewed global epidemic risk assessment frameworks and identified critical areas for improvement, particularly the need to incorporate nuanced, country-specific factors into pandemic preparedness plans to enhance their effectiveness in guiding national and global responses to future threats [2,11–13].

Here we aimed to capture the impact of the COVID-19 pandemic on key components of Vietnam's health system and the corresponding national responses. In doing so, we covered critical areas, including the health workforce, financing and resource mobilisation, governance, policies and service delivery structures, and the capacities of existing health information systems. By characterising the multifaceted impacts of the pandemic, we identified gaps within these components and proposed strategies to enhance the resilience and capacity of the health system to effectively manage future pandemics, whether they were emerging or re-emerging infectious diseases.

Prior to writing this viewpoint, we reviewed the available literature and evidence on the vulnerabilities and responses of Vietnam’s health system during the COVID-19 pandemic. For this purpose, we searched PubMed, Scopus, Google Scholar, and other databases using the following search string: (“COVID-19”[Mesh] OR “SARS-CoV-2”[Mesh] OR “Coronavirus”[Mesh] OR “COVID-19” OR “SARS-CoV-2” OR “Coronavirus”) AND (“Vietnam”[Mesh] OR “Vietnam” OR “Viet Nam”). We also searched for Vietnamese literature, including government reports, news, and papers related to the topics.

In reviewing these studies, we focused on thematic categories such as health system resilience, pandemic response strategies, the impact on HCWs, digital health adoption, and governance challenges. We categorised this information under key areas, including health workforce challenges, financial resource allocation, and the role of digital health interventions. Lastly, we created a narrative synthesis by integrating the data, highlighting areas of strength and vulnerability within Vietnam's health system, and proposing policy implications to strengthen future pandemic preparedness.

## HEALTH FINANCING

In Vietnam, the total health expenditure per capita is USD 166.2, accounting for 5.5% of the country’s gross domestic product, while 9.41% of the government’s total budget is allocated to health services. Approximately 90% of the population is covered by social health insurance; however, 43% of health expenses are paid out-of-pocket by individuals, indicating a significant personal financial burden and a high risk of experiencing catastrophic health expenditure [11]. Vietnam has established financing mechanisms for both epidemic preparedness (Level 4) and emergency response (Level 3) [12].

During the COVID-19 pandemic, the Vietnamese government implemented significant financial measures to support prevention and control efforts, including the establishment of the COVID-19 Vaccine Fund in May 2021, which was crucial for mobilising additional resources [13]. By December 2022, Vietnam had administered over 265 million doses of COVID-19 vaccines, achieving nearly 100% coverage for the first two doses among those aged 12 years and above.

The procurement process during the COVID-19 pandemic, however, faced several significant challenges [13]. First, the unpredictable and complex nature of the pandemic led to shortages in the supply of medical equipment, testing kits, and other necessary materials at various times [2]. Additionally, the sudden spike in demand caused sharp price increases, complicating the procurement efforts of local authorities and units. Most of the medical equipment, drugs, and vaccines had to be imported, resulting in delays, high costs, and a crisis-driven approach [14]. Some localities and units struggled with logistics and were hesitant to take proactive measures due to concerns about procurement processes.

## HEALTH WORKFORCE

Vietnam aims to distribute its healthcare workforce evenly nationwide, with over 400 000 HCWs and a target of 33 hospital beds and 15 doctors per 10 000 population by 2025. However, significant disparities persist, with urban areas like the Red River Delta having the highest workforce density, while rural regions such as the Mekong Delta and Central Highlands remain underserved [2]. During the COVID-19 pandemic, there has been a profound and multifaceted impact on HCWs [3]. These challenges included increased workloads, psychological stress, and physical health risks, compounded by inadequate protective equipment and inconsistent guidelines, while interacting enabling factors at multiple levels further contributed to severe stress, burnout, and decreased quality of life. It is estimated that about 40% of health workers experienced a significant prevalence of psychological disorders during the COVID-19 pandemic, especially those at the frontlines [15].

One of the critical issues faced by HCWs in Vietnam is the stigma associated with COVID-19, which has significant health implications. Those who underwent quarantine experienced substantial levels of stigma, which was strongly correlated with depression, anxiety, and stress [16]. A study in Da Nang City found that nearly half of the HCWs experienced increased stress levels, with severe stress being linked to longer working hours and direct contact with COVID-19 patients [17]. Similarly, HCWs at Dong Da and Dong Anh general hospitals reported high levels of anxiety and depression, with contributing factors such as human resources shortages and community discrimination [18]. Moreover, the pandemic has exposed the varying levels of health literacy and preparedness among HCWs. While there was generally high adherence to infection prevention measures, certain demographics, particularly those with higher education or white-collar jobs, perceived lower importance of some preventive measures, such as mandatory quarantine [19].

The pandemic's impact on the daily work and life of HCWs has also been significant. Research revealed that during the nationwide lockdown, many HCWs reported feeling undervalued and overworked, with significant regional disparities in their experiences. In central regions, HCWs were less likely to report discrimination but faced increased work pressure [20]. Additionally, the emotional toll of the pandemic on HCWs, especially those with less work experience or those working in high-risk departments, has been severe, necessitating the urgent need for psychological support and coping strategies [21].

## HEALTH INFORMATION SYSTEM

Vietnam has integrated health information and surveillance systems effectively, with data being used for planning at a central level and integrating human-animal-environmental factors [2]. Facility-based electronic health records are partially implemented, with ongoing efforts to expand coverage. Policy documents highlight both the challenges and resolutions in public-private data sharing and risk communication is facilitated through various channels, including telemedicine. These advancements are supported by initiatives such as the Ministry of Health's 2020–25 telemedicine programme, emphasising the government's commitment to enhancing healthcare accessibility and quality across the country [12].

The COVID-19 pandemic has highlighted the critical role of health information systems and digital health solutions in supporting Vietnam’s healthcare infrastructure. Health personnel in Vietnam have relied on various sources of COVID-19-related information, including mass media, online platforms, and peer educators. However, the accessibility of vital guidelines and prevention policies was comparatively low among certain groups, such as community health workers [22]. The need for improved dissemination strategies has become evident, pushing Vietnam to rethink its health communication efforts and ensuring that all HCWs are equipped with the essential knowledge to handle the pandemic effectively.

During the COVID-19 pandemic, Vietnam rapidly deployed several information technologies (IT) to enhance its healthcare system and improve its response to the crisis. These applications have included telehealth systems, digital health platforms, mobile apps, and artificial intelligence-driven solutions [23]. First, Vietnam leveraged telehealth extensively, especially through initiatives like the Remote Health Examination and Treatment project, which connected over 1000 healthcare facilities across the country. Telehealth allowed provincial hospitals to collaborate with central hospitals, facilitating consultations and treatments remotely. This reduced the strain on overcrowded hospitals and minimised patient travel and risk of infection [1]. However, the main challenges for this initiative included weak internet infrastructure in remote areas, inadequate IT literacy among HCWs, and the absence of standardised national regulations for telemedicine [2].

Second, Vietnam introduced several digital health applications to monitor and control the pandemic. These included the Bluezone app, which tracked close contacts with COVID-19 patients using Bluetooth technology, and electronic Communicable Disease Surveillance, which recorded case data and helped with contact tracing. Furthermore, the NCOVI app allowed citizens to report their health status, while the Vietnam Health Declaration app monitored travellers entering the country [24]. The pandemic also accelerated the implementation of telehealth systems at provincial, district, and commune levels. A notable example is the success of Phutho General Hospital in integrating telehealth technology to enhance healthcare delivery. This system proved particularly beneficial for rural patients who could access consultations from experienced doctors without travelling long distances [25].

Telemedicine, although introduced in Vietnam as early as 1998, saw a significant boost during the pandemic. Despite the effectiveness of various initiatives during the pandemic, telemedicine in Vietnam faces barriers such as inadequate infrastructure, limited IT literacy, and concerns over patient privacy [26]. The expansion of telemedicine requires a more structured approach, ensuring integration across all levels of the health system, particularly in resource-scarce settings. Further details on infrastructure requirements, such as robust digital connectivity and IT support, along with clear regulatory frameworks, are essential to assess the feasibility of telemedicine as a sustainable long-term solution.

## GOVERNANCE CAPACITY

Vietnam's governance capacity in public health has significantly improved in recent decades, particularly during emergencies like the COVID-19 pandemic. The country employed comprehensive data utilisation practices during the pandemic, including regular vulnerability assessments, to identify and protect high-risk populations and regions [1]. These assessments are crucial for shaping the government's response and ensuring that countermeasures are effectively deployed to mitigate the impacts of public health threats. Public trust in the healthcare system is actively measured through surveys, rapid assessments, and social listening [27]. This focus on assessing and maintaining trust helps ensure the success of health campaigns and encourages compliance with health guidelines, which are essential during crises like pandemics [28]. A clear and well-defined structure has also been established in the country, assigning specific roles and responsibilities to different agencies involved in public health [23,29]. This clarity in governance ensures that efforts are coordinated efficiently across all levels of government, from the central to the local, facilitating a unified response to health emergencies.

Moreover, Vietnam maintains an up-to-date national health emergency strategy, which is continuously refined based on new threats and insights from past experiences [1]. The country ensures that medical services are accessible, particularly in remote and underserved areas. The government also prioritises the allocation of financial and other resources, ensuring that healthcare facilities are equipped and staffed to meet both routine and emergency needs, reflecting a proactive and comprehensive approach to public health governance [14,30].

## OVERVIEW AND PREPAREDNESS FOR FUTURE PANDEMIC

The COVID-19 pandemic tested Vietnam’s health system across multiple areas: health financing, workforce management, information systems, and governance. Despite initial successes in controlling the virus through early lockdowns, vaccination campaigns, digital health tools, some systemic vulnerabilities still emerged. These included delays in medical supply procurement, uneven workforce distribution, and limitations in the country’s digital infrastructure. The health information system played a pivotal role in this sense, especially through telemedicine and contact tracing apps, though its reach was hindered by regional disparities. Therefore, while governance efforts were largely effective, the crisis exposed weaknesses in resource allocation and coordination.

Key areas for improvement include enhancing financial mechanisms to ensure swift resource disbursement during emergencies and localising the production of medical supplies to reduce reliance on imports. However, information on disparities in resource allocation between rural and urban healthcare responses is lacking. The health workforce remains unevenly distributed, with rural areas particularly underserved. Addressing this imbalance, providing better mental health support, and raising awareness for HCWs and managers, especially during crises, is therefore essential. Vietnam’s digital infrastructure, while showing promise through the novel telehealth platforms, requires significant investment to improve internet access in rural areas and integrate telemedicine into routine care. Lastly, governance must focus on better interagency coordination and developing clearer protocols for faster resource mobilisation, especially for remote regions.

In this viewpoint, we highlighted the critical need for Vietnam to adopt a more resilient, flexible health system for future pandemic preparedness. Enhancing health financing by establishing rapid-response financial frameworks will allow for more agile procurement of medical supplies and support to HCWs. Expanding telemedicine across rural areas will also be crucial in ensuring healthcare continuity during future outbreaks. Moreover, workforce planning must focus on equitable distribution of healthcare personnel and providing comprehensive psychological support. Strengthening governance structures and ensuring more efficient coordination across agencies can greatly improve crisis response efficiency.

Vietnam’s response to COVID-19 provides valuable insights into both the strengths and weaknesses of its health system. Early successes in pandemic control highlighted the effectiveness of decisive governance and societal cooperation. However, challenges such as delayed responses and gaps in evidence-informed policymaking underscore the need for systemic improvements. By addressing deficiencies in health financing, workforce distribution, digital infrastructure, and governance coordination, the country can strengthen its health system and improve preparedness for future public health crises.
